# Postoperative Morbidity of Dental Paediatric Patients Treated under General Anaesthesia at a University Hospital: An Observational Study

**DOI:** 10.1155/2022/9606010

**Published:** 2022-06-26

**Authors:** Lamis D. Rajab, Aseel E. Obaid, Mahmoud A. M. Hamdan, Yazan Hassona

**Affiliations:** ^1^Department of Paediatric and Preventive Dentistry, School of Dentistry, The University of Jordan, Amman, Jordan; ^2^Department of Oral and Maxillofacial Surgery, Oral Medicine, and Periodontics, School of Dentistry, The University of Jordan, Amman, Jordan

## Abstract

**Aims:**

To assess prevalence of postoperative morbidity signs and symptoms in children treated under GA, and to investigate the association between pre- and intra-operative factors with postoperative morbidity. *Study design* and

**Methods:**

Prospective, observational study supported by pre-operative, intra-operative, and postoperative questionnaire conducted on paediatric patients treated for dental reasons under general anaesthesia at a university hospital.

**Results:**

Hundred and fifty patients were included with average age 5.5 years. The majority (92%) experienced at least one postoperative morbidity sign and symptom on the day of treatment under GA and the majority of symptoms subside by the third day. Dental pain (81.3%), sleepiness (70%), and poor appetite (46.7%) were the most frequently reported. Logistic regression analysis showed that age, gender, type of treatment provided, use of LA, and duration of procedure were significantly associated with postoperative morbidity.

**Conclusions:**

Most patients experienced one or more morbidity sign/s or symptom/s during first postoperative day and the majority subsides by the third day. Dental pain and poor appetite were the most and long lasting reported. Factors that would predict the occurrence of postoperative morbidity were gender, patient's age, and duration of procedure.

## 1. Introduction

Behaviour guidance techniques have permitted the majority of paediatric dental patients to receive treatment in the dental clinic with minimal distress and without expressed fear. Minimal or moderate sedation has allowed others who are less amenable to receive treatment. In some children and individuals with special care needs who have extensive oral healthcare needs, acute situational anxiety, uncooperative age-appropriate behaviour, immature cognitive functioning, disabilities, or medical conditions various levels of sedation may be necessary to ensure compliance with dental treatment, including minimal sedation, moderate sedation, deep sedation, or general anaesthesia (GA) to receive dental treatment in a safe and humane fashion. [1, 2] There has been a change in recent years in the perception of professionals regarding the usefulness of general anaesthesia in children, both in young patients with extensive oral problems and/or difficult to manage behaviour and in disabled or medically compromised patients, as well as in oral surgery procedures. This kind of anaesthetic technique makes it possible to resolve all oral health problems in a single visit without the need for the child's cooperation [2, 3].

Despite all the benefits reported for treatment under GA, many problems and complications have also been reported. There is limited number of scientific publications on postoperative morbidity following GA. Reports of postoperative morbidities with paediatric dental rehabilitation under GA were found in the range of negligible to more than 90% of patients. [4–6] Postoperative morbidity signs and symptoms reported in children treated under GA included pain, nausea, vomiting, sore throat, sleepiness, and haemorrhage wound in the mouth. [5, 7–10] Postoperative mortality was also reported [2, 11].

Many factors were reported to trigger the development of postoperative complications such as dental procedure, patient age, patient medical status, premedication used, anaesthetic time, intubation difficulty, anaesthetic medications, and use of local anaesthesia (LA) and systemic analgesia intra-operatively [3–6, 12].

There is a lack of data regarding morbidity events related to GA for dentistry. A Medline search has shown that no studies have been conducted on postoperative morbidity following dental treatment under GA in children and this study was the first to investigate this issue in the country. Through this study, the authors sought to present a comprehensive assessment of postoperative morbidity in dental treatment under general anaesthesia in paediatric patient in a university hospital setting and to provide information relating to factors that would significantly predict postoperative morbidity in healthy children, which, to the best of the author's knowledge, was rarely discussed in the dental literature. Thus, the specific aims of the study were to assess the prevalence of postoperative morbidity in children following dental treatment under GA at a university hospital, to explore the commonly experienced postoperative symptoms in children undergoing dental treatment under GA, and to determine the relationship between pre- and intra-operative factors with postoperative morbidity signs and symptoms.

## 2. Materials and Methods

### 2.1. Ethical Approval

The study was approved by the Department of Paediatric Dentistry at the School of Dentistry and the Council of the School of Postgraduate Studies at the Jordan University Hospital. An IRB approval was also obtained (10/2017/18249) from the institutional review board at the Jordan University Hospital

An informed consent was obtained from the patients' legal guardians in the presence of one of the investigators (LDR and AEO) to finalize patient recruitment, explaining the aims, procedure, importance of the study, and reassurance regarding confidentiality of any information collected.

### 2.2. Study Design

This was a prospective observational study supported by a preformulated questionnaire. Data collection was carried out over a period of six months, from the beginning of October 2017 to the end of March 2018.

### 2.3. Population, Sampling Procedures, and Sample Size Calculation

Paediatric patients presenting to the specialty clinics of paediatric dentistry at XXX and scheduled for treatment under GA were selected, after matching the inclusion criteria. Review of the records of paediatric patients who attended for dental rehabilitation under GA at XXX in a period of 6 months showed that the total number of these patients was 138. Using the *G*-Power 3.0.10 sample size calculator (Faul et al.) [13] and utilizing statistics of multiple regression with moderate effect size of 0.5 at (*α* = 0.05) type level of significance and power of 0.8, acknowledging 11 predictors, estimated sample size was at least 123 subjects. However, a sample size of more than 123 sustains an increased power of the study (1-*α*) and overcome type I error.

### 2.4. The Questionnaire

The questionnaire used was divided into three parts.

#### 2.4.1. Pre-operative Questionnaire

The pre-operative questionnaire contained questions about demographic characteristics. It included information about the child's age and gender, insurance type, and level of education for both the mother and the father. Medical status was checked to ensure that the patient was medically fit. The questionnaire also included questions about the child's history of dental pain. This questionnaire was completed through interviewing the parents by one of the authors.

#### 2.4.2. Intra-operative Questionnaire

This part comprised dental procedure details. The operator, number of restorations, SSCs, pulpotomies, pulpectomies, and extractions, if performed, were recorded. Local anaesthesia (LA) if used and the number of carpules, duration of the dental procedure, and medications prescribed after recovery were recorded. This questionnaire was completed by AEO through observing and interviewing the paediatric dentist performing the dental procedure.

#### 2.4.3. Postoperative Questionnaire

Parents were contacted by phone calls on the day of dental treatment under GA (1st day), 2nd, 3rd, 5th, and a week after dental treatment. They were asked questions about their child's postoperative morbidity signs and symptoms, if present. Variables recorded were dental pain (no pain, mild, moderate, and severe), sleepiness, poor appetite, haemorrhage wound in the mouth, insomnia, sore throat, nausea, cough, fever, vomiting, and medication given for dental pain.

### 2.5. Inclusion and Exclusion Criteria

Paediatric patients classified as ASA I according to the American Society of Anaesthesiologists (ASA 2020) physical status classification system (American Society of Anaesthesiologists: ASA physical status classification system. [14] Accessed September 2020) who previously failed to undergo 1 successful dental treatment for reasons of poor cooperation or those with extensive treatment plan that required multiple appointments, and whose parents/caregivers accepted to be enrolled in the study and signed the consent form were included. Children whose parents did not sign the consent form, who were medically compromised, or lost to follow up after treatment, were excluded from the study.

### 2.6. Pilot Study

A pilot study was carried out on patients scheduled for dental treatment under GA before starting the main study to test the clarity of the questionnaire. A total of 10 parents who did not participate in the main study were invited to participate in the pilot study. Parents' ease of understanding and answering the questionnaire was satisfactory, and the pilot study indicated there was no need to change the proposed methods.

### 2.7. Drugs Used for GA and Method of Intubation

All patients were evaluated by the anaesthesiologists pre-operatively to ensure they are fit for GA. Anaesthetic protocols were standardized to reduce the risk of any bias or confounding factors. Nasal intubation was used for all patients. Induction of anaesthesia was performed through inhalation of sevoflurane. Opioid (Fentanyl) and muscle relaxant (rocuronium) were given intravenously. Isoflurane was given for the maintenance of anaesthesia. Dexamethasone was also given for all patients. Neostigmine and Atropine were given by the anaesthesiologists as reversal agents. Local anaesthetic (4% articaine with 1 : 100000 epinephrine) if used was administered according to the need estimated by the operating paediatric dentist and was always administered by infiltration technique. It was given to cases that required extraction of teeth to control pain and haemorrhage in case of extraction.

### 2.8. Treatment Provided

Dental treatments were performed by paediatric dentists at XXX. A throat pack and a mouth gag were used. Dental treatment completed for each paediatric patient was recorded and categorized into one or more of the following categories: restorations (composite or GI), SSC, extractions, pulpotomies, and/or pulpectomies. The order of the procedures was as follows: (1) fissure sealants, (2) restorative treatment (pulpotomies, root canals, and fillings), (3) and extraction. After the completion of the procedures for each quadrant, a topical fluoride treatment was applied.

### 2.9. Data Analysis

The statistical package for the Social Sciences (SPSS for Windows, version 21.0, SPSS Inc., and Chicago, IL, USA) was used to perform the statistical analysis of the collected data. Descriptive data included the prevalence of postoperative morbidity and commonly experienced postoperative symptoms by participants (frequency). Association and differences between the study variables and postoperative morbidity signs and symptoms were analysed using chi-squared test and Pearson correlation coefficient. The prediction of pre- and intra-operative factors on postoperative morbidity signs and symptoms was investigated using logistic regression analysis. Logistic regression analysis was used to investigate the simultaneous influence of different independent variables that had a statistically significant impact on morbidity signs and symptoms. For association, differences, and logistic regression, only Day 1 was included in the analysis. Days 2, 3, 5, and 7 were excluded from the analysis as postoperative morbidity signs and symptoms were observed mostly at Day 1. The level of significance for all tests was set at 5%.

## 3. Results

The total number of patients who were scheduled for treatment under GA during the study period was 162 patients. Among these, 8 patients were excluded from the study sample because they were medically compromised, 2 patients because they were lost to follow-up, and 2 others because they required the extraction of permanent first molars. The final study sample comprised 150 patients.

The average age of children who received dental treatment under GA was 5.5 years (SD ± 1.7) with ages ranging from 2.5 to 10.8 years. The number of male and female patients was almost equal (male 76 (50.7%) and female 74 (49.3%)). Of all patients, 72 (48%) had pre-operative dental pain, 60 (40%) had pain with associated swelling, and 18 (12%) had no history of pain.

The average number of treatments performed for each patient was 10.67 ± 4 with minimum treatments of 2 and maximum treatments of 22.  Table 1 shows the type of treatment performed under GA. The most commonly performed procedure under GA in the study sample was SSC (95.3%).

The number of patients who received LA during the procedure was 132 (88%). The number of LA carpules given per patient ranged from 0 to 2.5, with a mean of 1.0 (SD = ± 0.6). The duration of the dental procedure ranged between 20 and 180 minutes for the patient, with a mean of 72.9 (SD = ± 30.0) minutes.

Table 2 shows the prevalence of postoperative morbidity signs and symptoms at Day 1 (the day of dental treatment under GA), Day 2, Day 3, Day 5, and Day 7. Results demonstrated that 92% of all patients (*N* = 138) experienced at least 1 morbidity sign or symptom on the day of dental treatment under GA. On Day 1, the most frequently reported morbidity was dental pain (81.3%), followed by sleepiness (70%) and poor appetite (46.7%). Other symptoms experienced by the patients included haemorrhage (24.7%), insomnia (24.7%), and sore throat (20.7%). The least reported symptoms were nausea, cough, fever, and vomiting.

Also, Table 2 shows that On Day 2, the percentage of patients who experienced at least one symptom decreased to 70% where dental pain remained the leading reported complaint (59.3%), followed by poor appetite (23.3%), and sore throat (18%).

As shown in Table 2, on Day 3, 43% of patients were still experiencing signs and symptoms where dental pain (32%) and poor appetite (16.7%) continued to be the most commonly reported. On the fifth day, the percentage of patients who experienced signs and symptoms decreased to 31%, with only two symptoms reported: dental pain (30.7%) and poor appetite (6.7%). The percentage of patients who experienced symptoms during the seventh day was negligible being 2%. Symptoms reported were dental pain (1.3%) and poor appetite (1.3%).

Table 3 shows the significant effect of variables on postoperative morbidity signs and symptoms on Day 1. Postoperative dental pain and fever were reported more frequently among females (*P*=0.015), while there was no statistically significant difference among males and females for all the other recorded signs and symptoms. A statistically significant relation between developing postoperative nausea and history of pre-operative dental pain was found (*P*=0.016).

Children who received pulpotomy/pulpotomies had significantly more sore throat (*P*=0.039) and poor appetite (*P*=0.029) on Day 1, compared to those who did not receive this type of treatment. On the other hand, children who received extraction/s had a significantly higher percentage of reported haemorrhage (*P*=0.000) and nausea (*P*=0.031) on Day 1. Haemorrhage (*P*=0.01) and nausea (*P*=0.001) experienced on Day 1 were significantly higher among children who received LA intraoperatively as opposed to those who did not.

Pearson correlation coefficient showed that the age of the child had a statistically significant positive effect on reported nausea (*r* = 0.19, *P*=0.01) and vomiting (*r* = 0.24, *P*=0.004). However, age had a statistically significant negative effect on postoperative insomnia (*r* = −0.17, *P*=0.04). Also, Pearson correlation coefficient revealed that the duration of procedure had a significantly positive association with poor appetite (*r* = 21, *P*=0.04), and sleepiness experienced postoperatively (*r* = 19, *P*=0.02). The operator difference had no statistically significant effect on postoperative morbidity signs and symptoms experienced on the first day (*P* > 0.05).

### 3.1. Logistic Regression

See Table 4 shows the univariate unadjusted and multivariate adjusted logistic regression analysis of independent variables that demonstrated significant effects on Day 1. Gender and duration of procedure showed a statistically significant effect on reported dental pain. Females were 3 times more likely to have postoperative dental pain than males (OR = 3.05, 95% CI: 1.23, 7.54). Moreover, children were 2.6 more likely to have dental pain when the treatment duration exceeded 72 minutes.

Poor appetite reported on Day 1 was significantly affected by the duration of treatment and pulpotomy if was performed. Poor appetite was 0.37 less among children who received pulpotomy, compared to those who did not (OR = 0.37, 95% CI: 0.18, 0.75), and 2.5 times higher among those whose treatment duration exceeded 72 minutes (OR = 2.51, 95% CI: 1.23, 5.10).

The age of the child had a statistical significant effect on reporting sore throat on Day 1. Sore throat was 3.13 times more reported in children older than 5 (OR = 3.13, 95% CI: 1.23, 8.01). Reported nausea was also significantly affected by the age of the child. The odds of developing nausea were 3.28 higher when the age of the child was more than 5 years (OR = 3.28, 95% CI: 1.16, 9.29).

Reported fever was significantly affected only by gender. The odds of developing fever were 2.97 higher in female children (OR = 2.97, 95% CI: 1.08, 8.13).

The odds of developing vomiting on Day 1 were 1.51 higher if children underwent an extraction during their treatment under GA (OR = 1.51, 95% CI: 1.14, 2.02). Univariate logistic regression showed that extraction and LA had a statistically significant effect on reported haemorrhage. The odds of having haemorrhage on Day 1 could not be measured by logistic regression as haemorrhage was reported in all the cases requiring extraction and use of LA.

## 4. Discussion

This study describes the commonly experienced postoperative symptoms in children following dental treatment under GA at a university hospital and examined factors that might be related to postoperative morbidity to determine the significance of their effect on the morbidity of the paediatric patients.

Despite the importance of the topic, only few scientific, peer-reviewed articles addressed morbidity following comprehensive dental treatment under GA, many of which have demonstrated inconsistent results.

Most patients experienced at least one morbidity sign or symptom during the first postoperative day and the majority subsides by the third day. This was consistent with findings reported in previous studies. [3, 4, 8, 15] Costa et al. [9] reported that less than half of children experienced postoperative discomfort at the time of discharge. Enever et al. reported that morbidity signs and symptoms were negligible. [5] However, their study was based on patients' recall of postoperative symptoms, which raised a question about the possibility of a recall bias, as their surveys have been several months following the performed procedures.

Several postoperative symptoms were reported during the day of treatment under GA. Among these, the most commonly encountered symptoms were dental pain that required analgesia, followed by sleepiness and poor appetite. This is in accordance with results found by two previous studies. [8, 15] The sore throat and pain reported by the patients might have affected the children's ability to eat and could explain the high prevalence of poor appetite in the present study.

Upon reviewing each individual reported symptom, dental pain was the most commonly experienced and the longest-lasting postoperative symptom. This was consistent with the results reported in a few other studies. [3, 4, 8, 10, 12, 15] Vinckier et al. [6] found that pain was not reported as the most common postoperative morbidity in children treated under GA. However, all children in the sample were given pain medications at the end of treatment [6].

Sleepiness was the second most commonly encountered symptom during the first postoperative day, and it was rarely reported on the second day, and never on subsequent days. This finding was close to what was reported by Atan et al. who found that sleepiness was most commonly reported 1 hour after treatment and then decreased dramatically. [12] In our study, an opioid (fentanyl) and sevoflurane were given to all patients during the anaesthetic procedure. Reported postoperative sleepiness might be associated with the use of fentanyl and sevoflurane as sleepiness is considered one of their side effects. [16] The difference between studies in reporting morbidity might be because of the differences between the general anaesthetic or peri-operative analgesics regimens.

Unlike the other symptoms, poor appetite was reported in all days including the seventh day. The presence of dental pain until Day 7 may explain the reason behind the poor appetite. Alohali et al. believed that the “lack of appetite” was likely due to the children being cautious eating, while they have open and healing sockets [10].

Insomnia in this study decreased from the first day to the second and to the third postoperative day. According to Kain et al., alterations and other psychological changes are common in paediatric patients treated under GA and they are considered a part of the maladaptive postoperative responses that can be caused by different factors including the child's pre-operative anxiety [17].

In the present study, haemorrhage was reported on Day 1 in almost all cases that included extraction of teeth, but rarely on Day 2. Mayeda et al. found that haemorrhage was common after dental rehabilitation under GA and it subsides within 24 hours after the operation [18].

Other symptoms like sore throat, nausea, cough, and fever were reported less frequently in our study and were only experienced during the first 3 days. These findings are close to that found in previous studies [4, 6, 8, 15].

The least was vomiting, which was reported during the first two days by only a few of patients. The use of dexamethasone (corticosteroid) intra-operatively may account for the low occurrence of nausea, vomiting, and sore throat. Dexamethasone has been used routinely as an antiemetic medication in surgical patients and its effectiveness as a peri-operative agent has been well substantiated. [19] Dexamethasone has also been used to reduce and even prevent postoperative oedema, which results in sore throat experienced by the patient. [20] Sore throat, nausea, and vomiting were commonly reported during the first 24 hours in a previous study. [4] The prevalence of sore throat reported in this study is probably related to cases with difficult and traumatic intubation. The opioid (fentanyl) and sevoflurane used during the anaesthetic procedure may account for some of the nausea reported. Previous studies have reported that the use of opioids is related to increased postoperative nausea and vomiting. [8] The reported fever can be caused by tissue destruction, room temperature of the operating room, intra-operative medications used, dehydration, and bacteraemia. One cause of tissue destruction and bacteraemia is tooth extraction and oral surgery [3].

The percentage of patients who experienced morbidity in this study decreased gradually during the succeeding days after the procedure until it collectively reaches 2% in the seventh postoperative day. Needleman et al. found that symptoms were mostly reported on the first day, and they significantly decreased by the second and third day and ceased completely on the fourth and fifth day. [8] Farsi et al. observed that children regained their physical activity within the second postoperative day, and by the third day, a significant reduction or complete resolution of the symptoms was reported. [15] In our study, parents were asked about their children's reported symptoms only once on the day of dental treatment, 2nd, 3rd, 5th, and 7th day, whereas other studies reported symptoms at other different instances like at the recovery room, on the way back home, after reaching home, and on the first night. Consequently, the first day complaints were divided into shorter periods and hence lesser and different type of complaints.

Dental pain and fever were more commonly reported in female. According to Myles et al., the relationship between gender and postoperative morbidity could be due to the physiological differences between males and females and that females are known to express symptoms more often than males. [21] The association between gender and fever was, however, less easy to explain by the investigators. A previous study showed no significant association between fever elevation and gender. [15] An another study reported that the gender of the child was not associated with any postoperative morbidity [22].

In the present study, a history of pre-operative dental pain with or without a swelling or a draining fistula was found to be associated with reported nausea. This might be explained by the fact that most of the cases with a history of pain required extraction of teeth, which was associated with reported nausea. Costa et al. found a significant association between pre-operative pain score and postoperative discomfort reported by patients in the first week [9].

The age of the patient in this study was found to be positively associated with reported nausea and vomiting, and negatively associated with insomnia, which means that older children are more likely to report postoperative nausea and vomiting and less likely to report postoperative insomnia. Needleman et al. also found an association between patient's age and reported sleepiness and nausea. Children of older age may be more able to demonstrate their discomfort, whereas younger children rely on their parents' observation, which may be underestimated sometimes. [8] By contrast, Hu et al. observed that age was not related to any reported postoperative morbidity [22].

Among the treatments provided, only pulpotomy and extraction had shown to be associated with postoperative morbidity; that is, pulpotomy was associated with reported sore throat and poor appetite and extraction was significantly associated with reported haemorrhage and nausea. Dental pain was not found in our study to be associated with any type of treatment. This finding disagreed with the finding of previous studies. [8, 12, 15, 22] However, our finding was in agreement with the finding of Escanilla-Casal et al. [3] who concluded that postoperative complications were not related to the type and number of treatments performed, except in haemorrhage, which was found to be associated with extraction and oral surgery. Erkmen Almaz et al. reported that there was no significant relationship between postoperative dental pain and treatment type or number of teeth treated under GA [23].

Results of this study demonstrated that using LA during the procedure did not reduce postoperative dental pain. Several studies agreed that the use of LA was not related to reported dental pain. [24–27] Al-Bahlani et al. reported that the use of LA during treatment was not related to postoperative dental pain, and patients were found to be more distressed postoperatively. [28] In addition, it was found that the use of LA intra-operatively significantly reduced postoperative bleeding [26] and that children who received LA intra-operatively were in distress until anaesthesia wore off. [27] According to Townsend et al., sensation alteration in children is more distressing when it occurs in the facial area as the face is highly innervated, and thus, they are more aware of it. [27] However, two previous studies suggested that using LA during the procedure reduced postoperative dental pain, [29, 30] and Atan et al. reported a significant decrease in pain in patients who received LA. [12] Zhang et al. found no relationship between postoperative pain and dental bleeding and the number of extracted teeth and attributed their finding to the vasoconstrictor in the local anaesthesia used before extraction and the surgical filling of the alveolar socket after extraction [31].

In accordance with results of previous studies, our results confirmed that longer duration of treatment was significantly associated with postoperative appetite loss and sleepiness. [8, 12] By contrast, Hu et al. reported that the duration of the dental procedure was not significantly associated with postoperative morbidity [22].

It is recognized that certain limitations within this study could have affected the results. These limitations are inherent to observational study design that possibly could have been subject to information bias. Postoperative morbidity signs and symptoms were subjectively reported based on telephone calls with the parents. The postoperative signs and symptoms may have been misjudged by the parents. Also, postoperative pain experienced by patients was recorded by parental proxy. Proxy reporting of children morbidity may underestimate or overestimate the severity of a child's experienced symptom. Considering these limitations, the results should be interpreted cautiously. However, the study objectives, the quality of the methodology applied, the pilot study, and statistical tools may enable good and reliable control over these limitations.

It is noteworthy that this study has several strengths. One of the strengths of this study is that it represents a comprehensive examination and analysis of postoperative morbidity. Also, it was conducted at the largest referral hospital, located in the capital of the country, receiving patients from all over the country with different socioeconomic backgrounds. The large numbers of children in this study and the standardized protocols help to validate the results.

In the light of the present study, paediatric dentists should be aware of the possible occurrence of postoperative morbidity signs or symptoms so as to formulate a comprehensive, individualized management plan for each patient, and to counsel the parents about what to expect and how to react to observed postoperative morbidities. Also, understanding the factors that have a significant effect on postoperative morbidity in children enables paediatric dentists to better address these factors to prevent the occurrence of postoperative morbidities. Finally, paediatric patients should receive better postoperative pain management and the duration of treatment should be controlled to reduce the chance or severity of postoperative morbidity.

## 5. Conclusions

Most patients experience one or more morbidity sign/s or symptom/s during the first postoperative day and the majority subsides by the third day. The most common and long-lasting symptom was pain that required analgesia followed by poor appetite. Based on logistic regression analysis, the results of the present study propose that morbidity associated with the GA would be less of a problem than morbidity linked to dentistry. Factors that would significantly predict the occurrence of postoperative morbidity were gender, patient's age, and duration of procedure.

## Figures and Tables

**Table 1:** Type of treatment performed under GA.

Variables	Mean (SD)	Min–max
Restorations	2.21 (2.26)	0–10
Pulpotomy	1.41 (1.53)	0–6
Pulpectomy	0.03 (0.18)	0–1
Extraction	2.27 (2.16)	0–10
SSC	4.75 (2.18)	0–8

**Table 2:** Prevalence of postoperative morbidity signs and symptoms at Day 1 (the day of dental treatment under GA), then at Day 2, Day 3, Day 5, and Day 7.

Postoperative morbidity signs and symptoms	Day 1	Day 2	Day 3	Day 5	Day 7
	*N* (%)	*N* (%)	*N* (%)	*N* (%)	*N* (%)
Dental pain	122 (81.3)	89 (59.3)	48 (32)	46 (30.7)	2 (1.3)
Sleepiness	105 (70)	5 (3.3)	0 (0)	0 (0)	0 (0)
Poor appetite	70 (46.7)	35 (23.3)	25 (16.7)	10 (6.7)	2 (1.3)
Haemorrhage	37 (24.7)	5 (3.3)	0 (0)	0 (0)	0 (0)
Insomnia	37 (24.7)	16 (10.7)	3 (2)	0 (0)	0 (0)
Sore throat	31 (20.7)	27 (18)	14 (9.3)	0 (0)	0 (0)
Nausea	25 (16.7)	14 (9.3)	8 (5.3)	0 (0)	0 (0)
Cough	18 (12)	13 (8.7)	7 (4.7)	0 (0)	0 (0)
Fever	21 (14)	17 (11.3)	4 (2.7)	0 (0)	0 (0)
Vomiting	8 (5.3)	3 (2)	0 (0)	0 (0)	0 (0)
Need for analgesics	109 (72.7)	86 (57.3)	45 (30)	16 (10.7)	1 (0.7)

**Table 3:** Significant effect of variables on postoperative morbidity signs and symptoms on Day 1.

Postoperative morbidity signs and symptoms	Pre- and intra-operative variables *N* (%)					*P*
	Gender					
	Male	Female	—	—		
Dental pain	56 (45.9)	66 (54.1)				0.015
Fever	6 (28.6)	15 (71.4)				0.029

History of pain						
	No history of pain	Pain present	Pain with associated swelling or fistula			
Nausea	0 (0.0)	18 (72.0)	7 (28.0)			0.016

	Treatment provided					
	Pulpotomy			Extraction		
	Yes	No	*P*	Yes	No	*P*
Sore throat	13 (41.9)	18 (58.1)	0.039	—	—	—
Haemorrhage	—	—	—	37 (100)	0 (0.0)	0.000
Nausea				15 (60.0)	10 (40.0)	0.031

	Use of LA					
	Yes	No	—	—		
Haemorrhage	37 (100)	0 (0.0)				0.010

**Table 4:** Logistic regression analysis of independent variables that showed significance effects on day of treatment under GA.

Independent variables		Dependent variables	Absence/presence		Unadjusted odds ratio (95% CI)	*P*	Adjusted odds ratio (95% CI)	*P*
			No	Yes				
Age	<5 years	Sore throat	59	7	3.37 (1.35, 8.42)	0.009	3.13 (1.23, 8.01)	0.017
	>5 years		60	24				
	<5 years	Nausea	60	6	2.92 (1.09, 7.81)	0.032	3.28 (1.16, 9.29)	0.026
	>5 years		65	19				

Gender	Male	Dental pain	20	56	0.34 (0.14, 0.83)	0.015	3.05 (1.23, 7.54)	0.016
	Female		8	66				
	Male	Fever	70	6	2.97 (1.08, 8.13)	0.035	2.97 (1.08, 8.13)	0.035
	Female		59	15				

Pulpotomy	No	Poor appetite	26	35	0.48 (0.25, 0.93)	0.030	0.37 (0.18, 0.75)	0.006
	Yes		54	35				
	No	Sore throat	43	18	0.41 (0.18, 0.91)	0.029	0.60 (0.25, 1.44)	0.250
	Yes		76	13				

Extraction	No	Nausea	25	10	0.38 (0.15, 0.93)	0.035	0.86 (0.17, 4.31)	0.849
	Yes		100	15				
	No	Vomiting	35	0	1.51 (1.14, 2.02)	0.005	1.51 (1.14, 2.02)	0.005
	Yes		107	8				

LA use	No	Nausea	10	8	5.41 (1.88, 15.62)	0.002	5.23 (0.88, 31.32)	0.070
	Yes		115	17				

Duration	<72 min	Dental pain	20	61	2.50 (1.02, 6.11)	0.040	2.60 (1.04, 6.47)	0.040
	>72 min		8	61				
	<72 min	Poor appetite	49	32	1.88 (0.98, 3.60)	0.058	2.51 (1.23, 5.10)	0.011
	>72 min		31	38				

## Data Availability

The data that support the findings of this study are available from the corresponding author, [RLD], upon reasonable request.
